# The association between Dietary Oxidative Balance Score and muscle strength: NHANES 2011–2014

**DOI:** 10.3389/fnut.2025.1563451

**Published:** 2025-06-03

**Authors:** Li Sun, Changyu Xu, Gang Wang, Changming Jin, Haoran Hu, Yanyan Liu, Yang Li, Changhui Tang, Qingli Hua

**Affiliations:** ^1^Daqing Longnan Hospital, Daqing, China; ^2^Daqing People's Hospital, Daqing, China

**Keywords:** muscle strength, Dietary Oxidative Balance Score (DOBS), NHANES, physical activity, hypertension

## Abstract

**Background:**

Dietary Oxidative Balance Score (DOBS) is an indicator, and on muscle strength remains largely unexplored in existing literature. We aimed to investigate the potential association between DOBS and muscle strength, and to explore possible interactions between DOBS and other covariates.

**Methods:**

The association between DOBS and muscle strength under adjustment for confounders was analyzed using a weighted generalized linear regression model. A stratified analysis was used to conduct a sensitivity analysis of the linear regression.

**Results:**

The positive association between DOBS and muscle strength were found in this study. DOBS was positively associated with muscle strength only in the active activity subgroup. And the association between DOBS and muscle strength was stronger in the non-hypertensive group.

**Conclusions:**

DOBS and muscle strength showed an associated effect, and there was an interaction between DOBS with physical activity as well as hypertension, respectively. Individuals are encouraged to enhance DOBS levels. The findings may provide some potential theoretical references for the prevention of muscle strength loss through oxidative stress.

## 1 Introduction

Skeletal muscles, a vital body tissues, is essential for facilitating daily activities. Muscle loss, is characterized by a reduction in both mass and volume of skeletal muscle throughout the body (1), which may affect the ability of the body to move, its nutritional status, and its independence (2). Handgrip strength is a valid measure of muscle strength and can be easily assessed using hand dynamometers in clinical practice (3, 4). Several previous studies indicated that low handgrip strength was associated with increased all-cause and cardiovascular mortality (5), and was also significantly associated with a multitude of adverse health outcomes, including osteoporosis (6), cognitive decline (7), and diabetes (8). Handgrip strength may decline after middle age, and that decline accelerates with age (9). Therefore, the loss of muscle strength represented by low handgrip strength, particularly among older individuals, poses a significant public health concern in our aging society.

In recent years, a growing body of evidence has indicated the pivotal role played by oxidative stress mechanisms in the attenuation of muscle strength (10), which is defined as a state in which the cells and molecules in our body are exposed to excessive amounts of reactive oxygen species (11). Diet plays an integral part in obtaining nutrients and is a key factor influencing oxidative stress. For example, according to a review, the micro-nutrient zinc regulates the level of oxidative stress in the body (12), higher fat intake also may increase oxidative stress levels in the body leading to adverse outcomes (13). Epidemiological studies have shown that appropriate modification of dietary intake may prevent and treat diseases related to oxidative stress (14, 15). Due to the minor effect of a single diet on an individual's level of oxidative stress and the possibility of biological interactions involving multiple dietary oxidative components, assessing the individual effect of a single diet on exposures related to oxidative homeostasis was difficult. Dietary Oxidative Balance Score (DOBS) is an indicator calculated based on the oxidative effects of a wide range of nutrients, including 14 antioxidants and 2 pro-oxidants (16–18), which reflects the oxidative level of the diet in a more systematic and realistic way. In general, diets with higher DOBS typically include plenty of fruits, vegetables, nuts, and dietary fiber, which are components that contribute to lower levels of oxidative stress (19).

Several previous studies have shown that DOBS affects multiple adverse outcomes related to oxidative stress, such as osteoporosis (20), cancer (21), and all-cause mortality (17). The association between DOBS and muscle strength, however, has not been adequately investigated. Previous studies have shown that excessive oxidative stress markers such as reactive oxygen species (ROS) may impair mitochondrial function and reduce protein synthesis, which in turn leads to muscle atrophy and strength loss (22, 23). In addition, chronic inflammation due to oxidative stress promotes muscle catabolism and direct degradation of myofibrillar proteins (24). Higher levels of DOBS suggest that individuals consume antioxidant-based dietary patterns and may influence muscle strength by attenuating oxidative stress-induced muscle damage. Given the ability of DOBS to systematically assess the oxidative homeostatic properties of diets, exploring its association with muscle strength may not only deepen the understanding of the mechanisms of oxidative stress-mediated muscle decline, but also provide key evidence for the development of sarcopenia prevention strategies based on dietary interventions, especially in high-risk populations, which are of great clinical importance. We therefore aimed to investigate the potential association between DOBS and muscle strength using the National Health and Nutrition Examination Survey (NHANES).

## 2 Materials and methods

### 2.1 Sample

**Figure 1:**
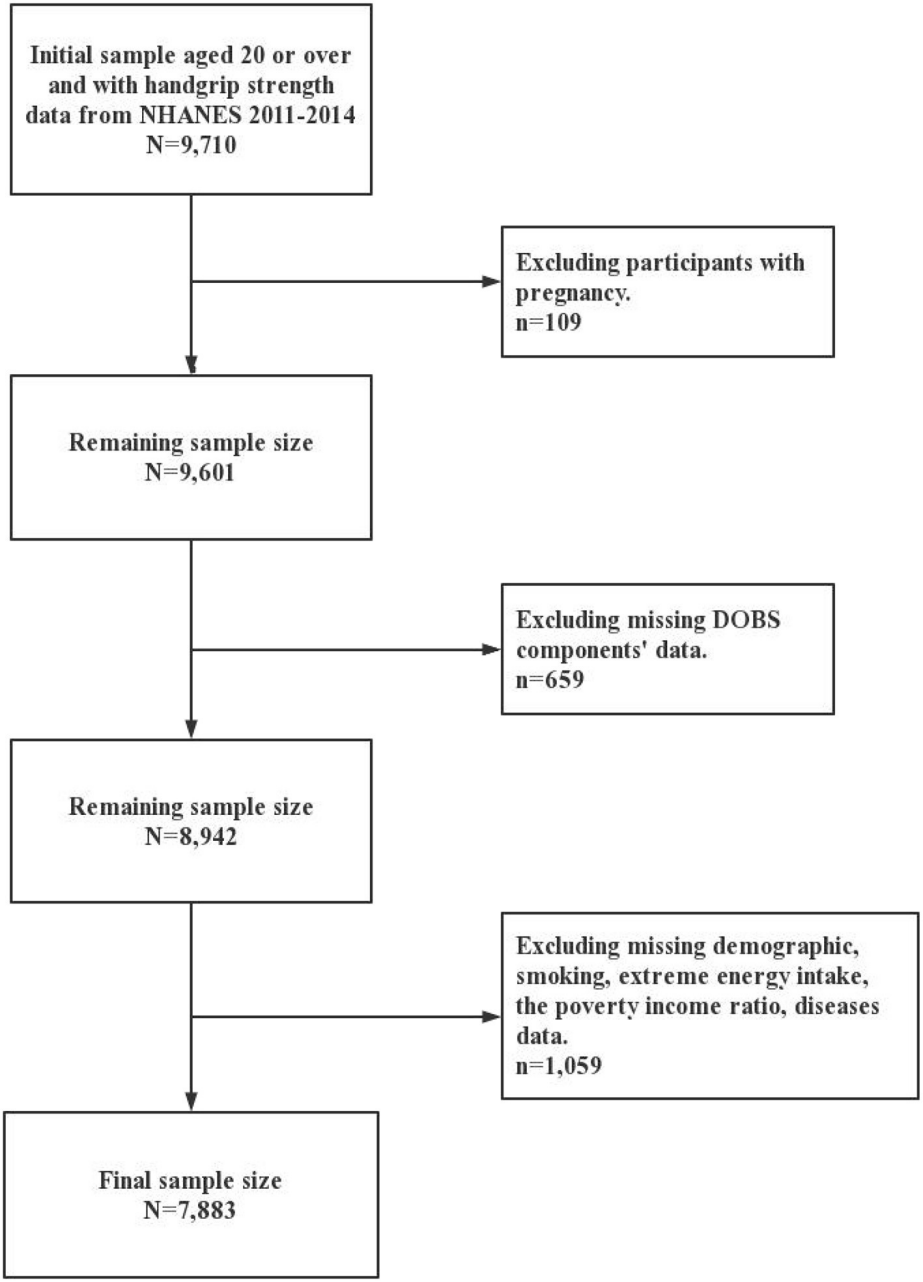
Flowchart for the study design and participants.

Participants in the present study were selected from the 2011 to 2014 cycle of the NHANES. It utilized stratified multi-stage probability sampling to collect information from relevant interviews, examinations, dietary questionnaires, and laboratory measurements. The NHANES data are publicly available at: https://www.cdc.gov/nchs/nhanes/index.htm. A total of 9,710 participants aged 20 or over and with complete data on handgrip strength were enrolled in two cycles of NHANES. Furthermore, 109 pregnant participants and 659 participants who missing DOBS data were excluded. Meanwhile, 1,059 participants were excluded due to extreme energy data (total energy intake <500 kcal/day or >5,000 kcal/day for females and <500 kcal/day or >8,000 kcal/day for males) and missing data on demographics, smoking, the poverty-income ratio (PIR), and related diseases (diabetes, hypertension, and arthritis). In this way the impact of unreliable dietary reporting was minimized and the completeness of adjustment for confounders was ensured. Ultimately, 7,883 participants were included in this study and the details were shown in Figure 1. The National Center for Health Statistics Research Ethics Review Board approved the NHANES study protocols. Written informed consent is obtained from all participants.

### 2.2 Muscle strength

Muscle strength was measured through a grip test using a handgrip Takei Dynamometer (TKK5401; Takei Scientific Instruments, Tokyo, Japan). Following a practice trial, participants were asked to forcefully squeeze the ergometer as hard as possible with one hand, breathing out while squeezing to avoid an increase in intrathoracic pressure. Then repeat the test on the other hand. A total of three tests were performed on each hand, alternating between the two hands between each test, with a 60-s rest period between measurements on the same hand. The combined grip strength was calculated as the sum of the largest reading from each hand, and expressed in kilogram. As the relationship between muscle strength and BMI is closely related (25), we used the relative grip strength calculated from the combined grip strength value to BMI ratio represents muscle strength (26). This adjustment accounts for body size differences and better reflects muscle function relative to metabolic demand, as validated in prior studies (27, 28). Higher relative grip strength values represented greater muscle strength of the participant.

### 2.3 Dietary Oxidative Balance Score

Two 24-h dietary recall interviews from NHANES were used for dietary antioxidant intake. The first face-to-face at a mobile examination center and the second by telephone 3–10 days later. Both recalls utilized the USDA Automated Multiple-Pass Method to ensure accuracy and minimize underreporting. Nutrient intake values were derived from the Food and Nutrient Database for Dietary Studies (FNDDS), which links food codes to standardized nutrient composition data. The mean value of the data was calculated and utilized as the dietary data for this study, as using non-consecutive days of dietary intake data was more accurate than using single-day data (29). On the basis of previous studies, the DOBS was composed of 16 dietary components (20, 30, 31). The two components, total fat and iron, were identified as pro-oxidants. And the 14 components, dietary fiber, carotene, riboflavin, niacin, calcium, magnesium, zinc, total folate, vitamins (B6, B12, C, and E), copper, and selenium, were identified as anti-oxidants. The DOBS assignment scheme was described in Supplementary Table S1. Each of the components was divided into three groups according to their tertiles. The scores for the first to third groups were 2, 1, and 0 for pro-oxidants, and the scores were 0, 1, and 2 for anti-oxidants, relatively. The DOBS obtained by summation was positively correlated with the total antioxidant capacity of the participants. Depending on the quartiles of the DOBS, we defined the first DOBS quartile as DOBS Q1 and correspondingly defined its second, third, and fourth quartiles as DOBS Q2, DOBS Q3 and DOBS Q4, respectively.

### 2.4 Covariate assessment

The selected covariates in this study were logically ordered and filtered based on existing literature and clinical experience. As mentioned previously, data on energy intake at extreme values were excluded. Race was categorized as non-Hispanic White and other race. Educational level was categorized as three groups: below high school, high school, and above high school. Marital status was categorized as married/living with a partner and Others (including widowed, divorced, separated, and never married). PIR was categorized as three groups: ≤ 1.30, 1.31–3.50, and >3.50. Serum cotinine levels was used to assess the extent of tobacco use and exposure to environmental tobacco smoke. And cotinine was categorized as three groups: ≤ 0.017 ng/mL, 0.018–0.239 ng/mL, and >0.239 ng/mL. Alcohol consumption was categorized as three groups: 0.0 g, 0.1–30.0 g, and >30.0 g. Physical activity (32) was categorized into two groups: the active group was defined as those who participated in more than 149 min of moderate physical activity, more than 74 min of vigorous physical activity, or more than 599 metabolic equivalents (MET) per week. The MET values were calculated based on the reported activity intensity classifications defined by NHANES, it classifies self-reported activity into predefined intensity levels (moderate/vigorous) based on participant descriptions: Active Work-Related Activity (High Intensity, 8.0), Moderate Work-Related Activity (Moderate Intensity, 4.0), Walking or Bicycling as a Means of Transportation (4.0), Vigorous Recreational PA (8.0), and Moderate Leisure Time PA (4.0). And the rest of the population was defined as the inactive group. If participants self-reported a physician's diagnosis of diabetes, hypertension, and arthritis, they were considered to have a history of these conditions.

### 2.5 Statistical analysis

In this study, all analyses considered sample weights as well as stratification and clustering methods to satisfy the requirements of the NHANES analysis guidelines for complex sampling designs. The continuous and categorical variables were described by means (standard errors) and quantities (percentages), respectively. To compare differences in categorical variables between different subgroups according to quartiles of DOBS, using the chi-square tests, while for continuous variables, using the one-way ANOVA. The association between DOBS and muscle strength under adjustment for confounders was analyzed using a weighted generalized linear regression model. Model 1 without adjustments; Model 2 adjusted for sex, age and race; And Model 3 additionally adjusted for energy intake, cotinine level, alcohol consumption, education level, marital status, physical activity, PIR, diabetes, hypertension and arthritis. A stratified analysis was used to conduct a sensitivity analysis of the linear regression. We conducted interaction effects by selecting covariates that were adjusted for in the model. Based on the regression model, an interaction term between DOBS and each covariate (e.g., DOBS × physical activity) was added to analyze the interaction, and then the R package “forestplot” was used to draw a forest plot for the hierarchical analysis of the interaction. All analyses were conducted using IBM SPSS 24.0 and R 4.2.1, and a two-sided *P*-value < 0.05 was considered significant. The statistical significance of interactions was evaluated using Wald tests, with a two-sided *P*-value < 0.05 indicating significant effect modification.

## 3 Results

Characteristics of the 7,883 participants included in the analyses are presented in Table 1, grouped according to the quartiles of DOBS. The participants included had a mean age of 46.71 years, with 50.49% being male, and a DOBS range of 2–31. Generally, it was observed that individuals with higher DOBS were predominantly male, Non-Hispanic White, and tended to be married or living with a partner (*P* < 0.05). Moreover, they exhibited higher energy intake and alcohol consumption, had advanced educational backgrounds, boasted a higher PIR, maintained lower levels of cotinine, and engaged in proactive physical activities (*P* < 0.05). Additionally, individuals with higher DOBS demonstrated a lower prevalence of both diabetes and arthritis (*P* < 0.05). Notably, participants in DOBS Q4 exhibited significantly higher handgrip strength compared to those in DOBS Q1.

**Table 1:** Demographic characteristics stratified by quartile of dietary oxidative balance score (*N*, %).

**Variables**	**Overall (*N* = 7,883)**	**DOBS Q1 (*N* = 2,051)**	**DOBS Q2 (*N* = 2,025)**	**DOBS Q3 (*N* = 2,081)**	**DOBS Q4 (*N* = 1,726)**	***P***
Age (year)	46.71 (0.51)	47.10 (0.72)	47.22 (0.58)	47.03 (0.82)	45.48 (0.80)	0.184
Energy intake (kcal/day)	2,130.50 (12.19)	1,435.30 (7.84)	1,852.44 (19.05)	2,250.78 (19.18)	2,913.67 (31.53)	**< 0.001**
Dietary oxidative balance score	16.68 (0.14)	7.03 (0.06)	13.69 (0.04)	19.49 (0.07)	25.37 (0.06)	**< 0.001**
Handgrip strength (kg/BMI)	2.66 (0.02)	2.30 (0.03)	2.53 (0.03)	2.69 (0.04)	3.08 (0.03)	**< 0.001**
Sex						**< 0.001**
Male	3,945 (50.49)	687 (31.23)	868 (42.25)	1,138 (53.50)	1,252 (73.11)	
Female	3,938 (49.51)	1,364 (68.77)	1,157 (57.75)	943 (46.50)	474 (26.89)	
Race						**< 0.001**
Non-Hispanic White	3,472 (67.68)	837 (61.52)	880 (66.61)	943 (71.22)	812 (70.46)	
Other race	4,411 (32.32)	1,214 (38.48)	1,145 (33.39)	1,138 (28.78)	914 (29.54)	
Education level						**< 0.001**
Below high school	1,515 (13.56)	546 (19.78)	397 (13.86)	331 (11.10)	241 (10.34)	
High school	1,724 (21.02)	528 (27.37)	449 (21.53)	416 (19.51)	331 (16.40)	
Above high school	4,644 (65.42)	977 (52.85)	1,179 (64.61)	1,334 (69.39)	1,154 (73.26)	
Marital status						**< 0.001**
Married/living with partner	4,580 (61.60)	1,062 (54.25)	1,140 (61.90)	1,290 (65.40)	1,088 (63.76)	
Others	3,303 (38.40)	989 (45.75)	885 (38.10)	791 (34.60)	638 (36.24)	
Poverty-income ratio						**< 0.001**
≤ 1.30	2,628 (24.18)	884 (34.97)	656 (23.44)	625 (20.71)	463 (18.96)	
1.31–3.50	2,724 (33.72)	741 (37.99)	702 (33.63)	720 (33.94)	561 (29.70)	
>3.50	2,531 (42.10)	426 (27.04)	667 (42.93)	736 (45.35)	702 (51.34)	
Cotinine (ng/mL)						**< 0.001**
≤ 0.017	2,697 (38.82)	545 (29.26)	695 (39.08)	816 (42.22)	641 (43.47)	
0.018–0.239	2,562 (29.24)	636 (28.33)	665 (28.72)	673 (29.92)	588 (29.86)	
>0.239	2,624 (31.94)	870 (42.41)	665 (32.20)	592 (27.86)	497 (26.67)	
Alcohol consumption (g)						**< 0.001**
0.0	5,557 (66.07)	1,617 (76.86)	1,460 (66.82)	1,404 (62.10)	1,076 (59.90)	
0.1–30.0	1,546 (21.80)	310 (15.22)	381 (20.29)	430 (24.22)	425 (26.66)	
>30.0	780 (12.13)	124 (7.92)	184 (12.89)	247 (13.68)	225 (13.44)	
Physical activity						**< 0.001**
Inactive	3,008 (34.84)	957 (43.92)	803 (34.81)	766 (36.03)	482 (25.30)	
Active	4,875 (65.16)	1,094 (56.08)	1,222 (65.19)	1,315 (63.97)	1,244 (74.70)	
Diabetes						**0.007**
Yes	959 (9.06)	308 (11.43)	240 (8.03)	261 (9.99)	150 (6.95)	
No	6,924 (90.94)	1,743 (88.57)	1,785 (91.97)	1,820 (90.01)	1,576 (93.05)	
Hypertension						0.112
Yes	2,822 (32.40)	830 (34.48)	712 (32.31)	734 (34.08)	546 (28.76)	
No	5,061 (67.60)	1,221 (65.52)	1,313 (67.69)	1,347 (65.92)	1,180 (71.24)	
Arthritis						**0.002**
Yes	1,992 (24.05)	617 (27.86)	527 (24.71)	487 (25.01)	361 (18.84)	
No	5,891 (75.95)	1,434 (72.14)	1,498 (75.29)	1,594 (74.99)	1,365 (91.16)	

Continuous variables were expressed with means (standard error) and categorical variables were described with quantities (percentages). Continuous variables were compared in four groups using One-Way ANOVA and categorical variables were compared using chi-square tests.

P < 0.05 was set as the threshold of statistical significance and marked in bold values.

Table 2 showed the results of linear regression models of DOBS on relative handgrip strength in different models. We found that DOBS (continuous value) was significantly positively associated with handgrip strength in all three models (*P* < 0.05). In the fully adjusted model (Model 3), the β and 95%CI of the DOBS Q3 and DOBS Q4 groups were 0.078 (0.005, 0.150) and 0.161 (0.059, 0.262) compared with the DOBS Q1 group (*P* < 0.05), respectively.

**Table 2:** Linear regression model of dietary oxidative balance score on handgrip strength.

**Variables**	**Model 1**		**Model 2**		**Model 3**	
	β **(95% CI)**	***P***	β **(95% CI)**	***P***	β **(95% CI)**	***P***
Dietary oxidative balance score (continuity value)	0.040 (0.036, 0.044)	**< 0.001**	0.012 (0.009, 0.016)	**< 0.001**	0.007 (0.002, 0.013)	**0.006**
DOBS Q1	1.000 (Ref)	-	1.000 (Ref)	-	1.000 (Ref)	-
DOBS Q2	0.231 (0.156, 0.306)	**< 0.001**	0.100 (0.040, 0.160)	**0.002**	0.044 (−0.018, 0.106)	0.158
DOBS Q3	0.393 (0.307, 0.480)	**< 0.001**	0.123 (0.054, 0.192)	**0.001**	0.078 (0.005, 0.150)	**0.036**
DOBS Q4	0.778 (0.697, 0.860)	**< 0.001**	0.258 (0.195, 0.322)	**< 0.001**	0.161 (0.059, 0.262)	**0.003**

Model 1 without adjustments; Model 2 additionally adjusted for sex, age and race; Model 3 additionally adjusted for energy intake, cotinine level, alcohol consumption, education level, marital status, physical activity, PIR, diabetes, hypertension, and arthritis. The bold indicates a statistically significant *P*-value of < 0.05.

**Figure 2:**
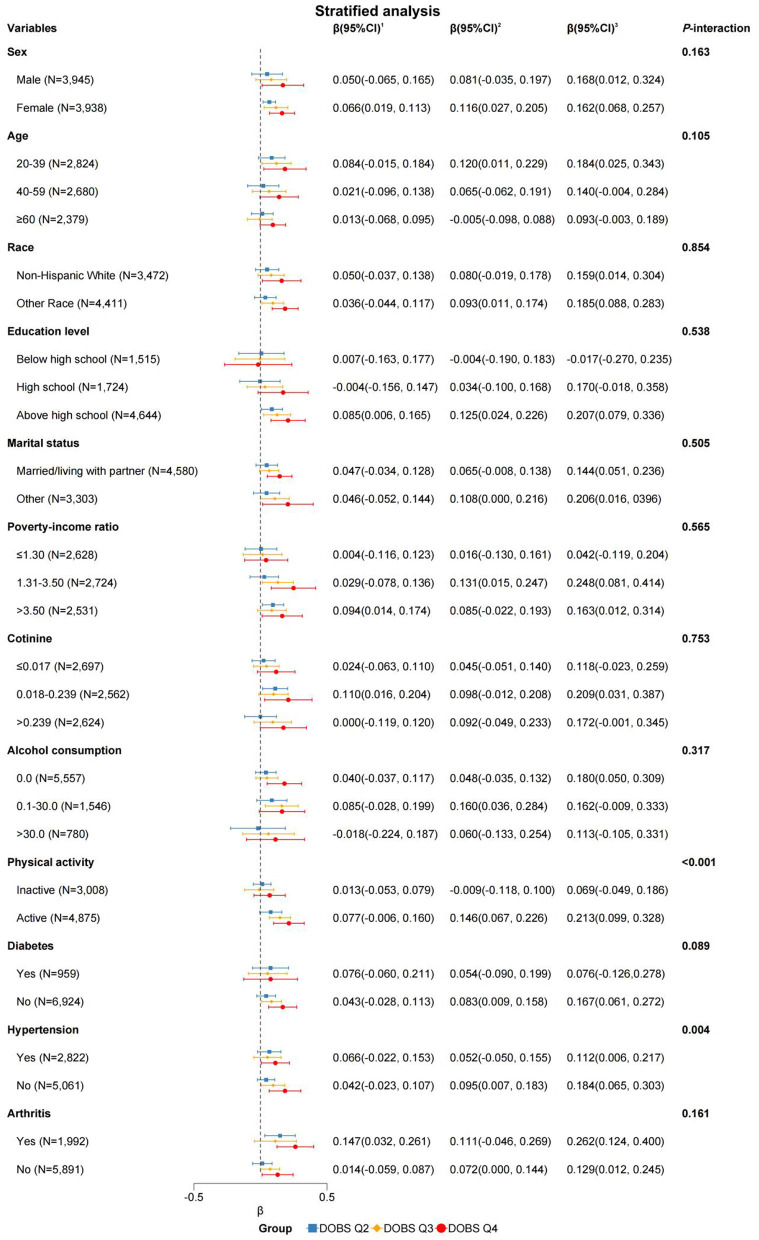
Forest plot of stratified analysis of the associations between dietary oxidative balance score and handgrip strength. Adjusted for sex, age, race, energy intake, cotinine level, alcohol consumption, education level, marital status, physical activity, PIR, diabetes, hypertension, and arthritis. ^1^DOBS Q2, ^2^DOBS Q3, ^3^DOBS Q4. Of note, the variables examined in this table were not adjusted.

To validate the robustness of our findings against potential confounders, subgroup analyses and interaction tests were conducted based on sex, age, race, education level, marital status, PIR, cotinine level, alcohol consumption, physical activity, and the presence of three specific diseases. Interaction tests revealed statistically significant effect modification by physical activity (*P* < 0.001 for DOBS × physical activity interaction) and hypertension status (*P* = 0.004 for DOBS × hypertension interaction) by Wald tests, as depicted in Figure 2. The results in Table 3 indicate a significant association between DOBS and handgrip strength in the active physical activity subgroup. Specifically, in the active group, the β and 95%CI of the DOBS Q3 and DOBS Q4 groups respectively were 0.146 (0.067, 0.226) and 0.213 (0.099, 0.328) in the final model (Model 3). However, no significant association was found among inactive individuals. Additionally, the association between DOBS and handgrip strength was more pronounced in non-hypertensive individuals, as shown in Table 4. In the final model (Model 3), the β and 95%CI of the DOBS Q3 and DOBS Q4 groups respectively were 0.095 (0.007, 0.183) and 0.184 (0.065, 0.303) in the subgroup of non-hypertension, and the β and 95%CI of the DOBS Q4 group was 0.112 (0.006, 0.217) in hypertension subgroup compared with DOBS Q1 group (*P* < 0.05).

**Table 3:** Stratified analysis of the associations between dietary oxidative balance score and handgrip strength by physical activity.

**Variables**	**Model 1**		**Model 2**		**Model 3**	
	β **(95% CI)**	***P***	β **(95% CI)**	***P***	β **(95% CI)**	***P***
**Inactive**					
DOBS Q1	1.000 (Ref)	-	1.000 (Ref)	-	1.000 (Ref)	-
DOBS Q2	0.177 (0.053, 0.302)	**0.007**	0.068 (0.002, 0.134)	**0.044**	0.013 (−0.053, 0.079)	0.696
DOBS Q3	0.204 (0.056, 0.353)	**0.008**	0.008 (−0.106, 0.121)	0.891	−0.009 (−0.118, 0.100)	0.867
DOBS Q4	0.510 (0.358, 0.663)	**< 0.001**	0.123 (0.009, 0.236)	**0.035**	0.069 (−0.049, 0.186)	0.242
DOBS (continuity value)	0.023 (0.015, 0.031)	**< 0.001**	0.003 (−0.003, 0.009)	0.289	0.000 (−0.006, 0.006)	0.951
**Active**					
DOBS Q1	1.000 (Ref)	-	1.000 (Ref)	-	1.000 (Ref)	-
DOBS Q2	0.211 (0.097, 0.325)	**0.001**	0.100 (0.017, 0.182)	**0.020**	0.077 (−0.006, 0.160)	0.069
DOBS Q3	0.457 (0.352, 0.562)	**< 0.001**	0.181 (0.114, 0.249)	**< 0.001**	0.146 (0.067, 0.226)	**0.001**
DOBS Q4	0.783 (0.687, 0.879)	**< 0.001**	0.281 (0.212, 0.349)	**< 0.001**	0.213 (0.099, 0.328)	**0.001**
DOBS (continuity value)	0.042 (0.038, 0.047)	**< 0.001**	0.015 (0.011, 0.018)	**< 0.001**	0.011 (0.006, 0.017)	**< 0.001**

Model 1 without adjustments; Model 2 additionally adjusted for sex, age, and race; Model 3 additionally adjusted for energy intake, cotinine level, alcohol consumption, education level, marital status, PIR, diabetes, hypertension, and arthritis. The bold indicates a statistically significant *P*-value of < 0.05.

**Table 4:** Stratified analysis of the associations between dietary oxidative balance score and handgrip strength by presence or absence of hypertension.

**Variables**	**Model 1**		**Model 2**		**Model 3**	
	β **(95% CI)**	***P***	β **(95% CI)**	***P***	β **(95% CI)**	***P***
**Hypertension**					
DOBS Q1	1.000 (Ref)	-	1.000 (Ref)	-	1.000 (Ref)	-
DOBS Q2	0.316 (0.171, 0.460)	**< 0.001**	0.123 (0.042, 0.205)	**0.004**	0.066 (−0.022, 0.153)	0.138
DOBS Q3	0.417 (0.284, 0.549)	**< 0.001**	0.088 (0.006, 0.170)	**0.035**	0.052 (−0.050, 0.155)	0.306
DOBS Q4	0.743 (0.608, 0.878)	**< 0.001**	0.169 (0.090, 0.247)	**< 0.001**	0.112 (0.006, 0.217)	**0.039**
DOBS (continuity value)	0.036 (0.030, 0.043)	**< 0.001**	0.007 (0.003, 0.011)	**0.001**	0.004 (−0.002, 0.010)	0.176
**Non-hypertension**				
DOBS Q1	1.000 (Ref)	-	1.000 (Ref)	-	1.000 (Ref)	-
DOBS Q2	0.175 (0.081, 0.268)	**0.001**	0.084 (0.019, 0.149)	**0.013**	0.042 (−0.023, 0.107)	0.201
DOBS Q3	0.378 (0.269, 0.487)	**< 0.001**	0.139 (0.054, 0.224)	**0.002**	0.095 (0.007, 0.183)	**0.035**
DOBS Q4	0.753 (0.655, 0.852)	**< 0.001**	0.279 (0.201, 0.358)	**< 0.001**	0.184 (0.065, 0.303)	**0.003**
DOBS (continuity value)	0.040 (0.035, 0.045)	**< 0.001**	0.014 (0.010, 0.018)	**< 0.001**	0.009 (0.003, 0.016)	**0.007**

Model 1 without adjustments; Model 2 additionally adjusted for sex, age and race; Model 3 additionally adjusted for energy intake, cotinine level, alcohol consumption, education level, marital status, physical activity, PIR, diabetes, and arthritis. The bold indicates a statistically significant *P*-value of < 0.05.

In order to further analyze the effect of DOBS combined with physical activity on handgrip strength, we divided the participants into eight subgroups based on the interaction, as shown in Supplementary Table S2. We used the subgroup with DOBS Q4 and physically active (subgroup 1) as the reference group, as it represents the combination of the highest dietary antioxidant level with the best physical activity state, expected to be ideal for muscle strength. Compared with the subgroup 1 (DOBS Q4 and the active activity group), all subgroups showed a statistically significant and negative association with handgrip strength (*P* < 0.05), except for the subgroup 2 (DOBS Q3 and the active activity group). Similarly, we also categorized the participants into eight subgroups based on DOBS and hypertension as shown in Supplementary Table S3. The results showed that all other subgroups were statistically significantly negatively associated with handgrip strength compared to subgroup 1 (DOBS Q4 and the non-hypertension group; *P* < 0.05).

## 4 Discussion

In the present study, we explored the potential association between DOBS and muscle strength as represented by handgrip strength based on NHANES and found a positive association between DOBS and grip strength after adjusting for a multitude of confounders. In addition, there were interactions between DOBS with physical activity and hypertension, respectively. The findings may provide some potential theoretical references for the prevention of muscle strength loss through oxidative stress.

On the best of our knowledge, this is the first study to explore the potential association between DOBS and muscle strength. The results showed a positive association between DOBS, which represents the antioxidant capacity of dietary sources, and muscle strength. It was consistent with the findings of previous studies. A review study showed that dietary supplementation with antioxidant properties may reduce muscle loss symptoms (33). Muscle strength and function could be effectively improved by consuming antioxidant components such as multivitamins and trace minerals such as magnesium (34). The following related mechanisms provide further support for the findings of this study. Dietary intake could influence the level of oxidative stress and inflammation in the body (35), and ROS and accumulation of inflammatory factors have been implicated as potential mechanisms for loss of muscle strength (23, 36). ROS are generated by adding an electron to an oxygen molecule and exists in almost all tissues of the organism. This reactive element is quite harmful and leads to oxidative stress, thus damaging other components such as DNA, proteins, and lipids, which then causes damage to cells and tissues (37, 38). The loss of muscle strength is precisely due to the overproduction of ROS in the organism, especially in skeletal muscle, and the reduced capacity of the antioxidant system (39). The results suggest that with a higher intake of antioxidant dietary components, it may be possible to reduce the harm caused to the organism by the overproduction of ROS, thus protecting muscle strength from damage.

Besides, oxidative stress is closely associated with inflammation and exerts a pro-inflammatory effect through activation of nuclear factor-kappa B, which increases the expression of chemokines and cytokines (24, 40). Several previous studies have demonstrated that higher levels of inflammatory markers, such as TNF-α, and IL-6, are associated with lower muscle strength (41, 42). These inflammatory factors may stimulate the loss of proteins in muscle cells and induce protein denaturation, leading to a reduction in muscle strength (22, 43). Similarly, elevated levels of DOBS may mitigate the decline in muscle strength induced by inflammation, thereby positively influencing muscle strength. Thus, oxidative stress and resulting inflammation play a pivotal role in muscle strength and could be crucial targets for the development of dietary strategies aimed at preventing sarcopenia.

People of all ages, young and old, are known to benefit from regular physical activity. Sedentary lifestyles are risk factors for muscle weakness, and physical activity has been shown to increase the body's aerobic capacity, muscle strength and endurance (44, 45). Our findings suggested an interaction between DOBS and physical activity. DOBS was positively associated with muscle strength only in the active subgroup and not significantly in the inactive. It also suggested that dietary intake with higher antioxidant capacity may increase the positive effects of active physical activity on muscle strength. For example, vitamin C and E supplementation has been shown to reduce exercise-induced oxidative stress and improve muscle recovery (46). Physical activity has frequently been positively associated with healthy dietary intake, with the active-body group's eating habits being closer to the recommended healthy eating guidelines. And active individuals often exhibit higher metabolic rates and nutrient absorption efficiency. Meanwhile, those who increased their physical activity were more likely to report improvements in diet quality (47, 48). This behavioral synergy may create a compounding effect, where DOBS and physical activity jointly mitigate oxidative stress and inflammation, thereby preserving muscle strength.

Our data revealed that there is an association between DOBS and hypertension. The association between DOBS and muscle strength was stronger in the non-hypertensive group than in the hypertensive group, and DOBS paired with non-hypertension revealed a significant positive association with muscle strength. A large cohort research in France (49) found that eating a diet high in antioxidants may lower the chance of developing hypertension and the pathological damage caused by hypertension. Furthermore, given there is a link between hypertension and low levels of muscle strength (50), DOBS may improve muscle strength by avoiding the establishment of hypertension and thus muscle strength. In the hypertensive population, however, the pathophysiology appeared to dampen the beneficial benefits of DOBS on muscle strength. Hypertension is characterized by elevated systemic oxidative stress and chronic inflammation, may impairs endothelial function and microvascular perfusion. These processes may overwhelm dietary antioxidant defenses (reflected by DOBS), reducing their capacity to protect skeletal muscle from oxidative damage. To lower the risk of sarcopenia in the hypertension group compared to the non-hypertensive group, a higher antioxidant diet is required.

This study has several advantages. First, this analysis employed NHANES data weighted for complexity, which is more typical of US population health. Second, we employed DOBS to analyze effects on muscle strength's oxidative potential while simultaneously accounting for numerous dietary components. Third, this is the first study to look at the link between DOBS and muscle strength. The study, however, still had some limitations. First, our individual NHANES data may be prone to recall and selection bias. Second, because this was a cross-sectional study, determining a causal association between DOBS and muscle strength is difficult. Besides, detailed training variables were unavailable in NHANES. Future studies incorporating device-measured PA could better disentangle the effects of training regimens on the DOBS and muscle strength relationship. Although our findings suggest the link between DOBS and muscle strength, randomized controlled trials are essential to confirm causality. The precise nature of their interaction will need to be examined further in the future.

## 5 Conclusion

In this cross-sectional study, DOBS and muscle strength showed an associated positive effect, and there was an interaction between DOBS with physical activity as well as hypertension, respectively. Our findings suggest that maintaining higher DOBS levels through antioxidant-rich dietary patterns may warrant further investigation for populations at risk of muscle strength decline. Further longitudinal or interventional studies are warranted to explore the potential role of DOBS in mitigating muscle deterioration. The findings may provide some potential theoretical references for the prevention of muscle strength loss through oxidative stress.

## Data Availability

The datasets presented in this study can be found in online repositories. The names of the repository/repositories and accession number(s) can be found below: the dataset supporting the conclusions of this article is available in the National Health and Nutrition Examination Survey (NHANES), hyperlink to dataset in https://www.cdc.gov/nchs/nhanes/index.htm.

## References

[1] Cruz-Jentoft AJ Sayer AA. Sarcopenia. Lancet. (2019) 393:2636–46. 10.1016/S0140-6736(19)31138-9 31171417

[2] Studenski SA Peters KW Alley DE Cawthon PM McLean RR Harris TB. The FNIH sarcopenia project: rationale, study description, conference recommendations, and final estimates. J Gerontol A Biol Sci Med Sci. (2014) 69:547–58. 10.1093/gerona/glu010 24737557 PMC3991146

[3] Lunt E Ong T Gordon AL Greenhaff PL Gladman JRF. The clinical usefulness of muscle mass and strength measures in older people: a systematic review. Age Ageing. (2021) 50:88–95. 10.1093/ageing/afaa123 32706848 PMC7793605

[4] Soysal P Hurst C Demurtas J Firth J Howden R Yang L. Handgrip strength and health outcomes: umbrella review of systematic reviews with meta-analyses of observational studies. J Sport Health Sci. (2021) 10:290–5. 10.1016/j.jshs.2020.06.009 32565244 PMC8167328

[5] Celis-Morales CA Welsh P Lyall DM Steell L Petermann F Anderson J. Associations of grip strength with cardiovascular, respiratory, and cancer outcomes and all cause mortality: prospective cohort study of half a million UK Biobank participants. BMJ. (2018) 361:k1651. 10.1136/bmj.k1651 29739772 PMC5939721

[6] Cheung CL Tan KC Bow CH Soong CS Loong CH Kung AW. Low handgrip strength is a predictor of osteoporotic fractures: cross-sectional and prospective evidence from the Hong Kong Osteoporosis Study. Age. (2012) 34:1239–48. 10.1007/s11357-011-9297-2 21853264 PMC3448988

[7] Yang L Koyanagi A Smith L Hu L Colditz GA Toriola AT. Hand grip strength and cognitive function among elderly cancer survivors. PLoS ONE. (2018) 13:e0197909. 10.1371/journal.pone.0197909 29864112 PMC5986134

[8] Karvonen-Gutierrez CA Peng Q Peterson M Duchowny K Nan B Harlow S. Low grip strength predicts incident diabetes among mid-life women: the Michigan Study of Women's Health Across the Nation. Age Ageing. (2018) 47:685–91. 10.1093/ageing/afy067 29726885 PMC6108393

[9] Bohannon RW. Hand-grip dynamometry predicts future outcomes in aging adults. J Geriatr Phys Ther. (2008) 31:3–10. 10.1519/00139143-200831010-00002 18489802

[10] Howard C Ferrucci L Sun K Fried LP Walston J Varadhan R. Oxidative protein damage is associated with poor grip strength among older women living in the community. J Appl Physiol (1985). (2007) 103:17–20. 10.1152/japplphysiol.00133.2007 17379753 PMC2646087

[11] Sies H. Oxidative stress: oxidants and antioxidants. Exp Physiol. (1997) 82:291–5. 10.1113/expphysiol.1997.sp004024 9129943

[12] Gammoh NZ Rink L. Zinc in infection and inflammation. Nutrients. (2017) 9:624. 10.3390/nu9060624 28629136 PMC5490603

[13] Tan BL Norhaizan ME. Effect of high-fat diets on oxidative stress, cellular inflammatory response and cognitive function. Nutrients. (2019) 11:12579. 10.3390/nu11112579 31731503 PMC6893649

[14] Food as medicine: translating the evidence. Nat Med. (2023) 29:753–4. 10.1038/s41591-023-02330-7 37041384

[15] Fontana L Partridge L. Promoting health and longevity through diet: from model organisms to humans. Cell. (2015) 161:106–18. 10.1016/j.cell.2015.02.020 25815989 PMC4547605

[16] Zhang W Peng SF Chen L Chen HM Cheng XE Tang YH. Association between the oxidative balance score and telomere length from the National Health and Nutrition Examination Survey 1999-2002. Oxid Med Cell Longev. (2022) 2022:1345071. 10.1155/2022/1345071 35186180 PMC8850082

[17] Liu J Wang W Wen Y. Association of dietary oxidative balance score and sleep duration with the risk of mortality: prospective study in a representative US population. Public Health Nutr. (2023) 13:1–10. 10.1017/S1368980023001155 37309207 PMC10564614

[18] Goodman M Bostick RM Dash C Flanders WD Mandel JS. Hypothesis: oxidative stress score as a combined measure of pro-oxidant and antioxidant exposures. Ann Epidemiol. (2007) 17:394–9. 10.1016/j.annepidem.2007.01.034 17462547

[19] Wang X Hu J Liu L Zhang Y Dang K Cheng L. Association of dietary inflammatory index and dietary oxidative balance score with all-cause and disease-specific mortality: findings of 2003-2014 National Health and Nutrition Examination Survey. Nutrients. (2023) 15:3148. 10.3390/nu15143148 37513566 PMC10383761

[20] Wang J Xing F Sheng N Xiang Z. Associations of dietary oxidative balance score with femur osteoporosis in postmenopausal women: data from the National Health and Nutrition Examination Survey. Osteoporos Int. (2023) 34:2087–100. 10.1007/s00198-023-06896-3 37648795

[21] Mao Z Prizment AE Lazovich D Gibbs DC Bostick RM. Dietary and lifestyle oxidative balance scores and incident colorectal cancer risk among older women; the Iowa women's health study. Nutr Cancer. (2021) 73:2323–35. 10.1080/01635581.2020.1821904 32981353

[22] Li YP Reid MB. NF-kappaB mediates the protein loss induced by TNF-alpha in differentiated skeletal muscle myotubes. Am J Physiol Regul Integr Comp Physiol. (2000) 279:R1165–70. 10.1152/ajpregu.2000.279.4.R1165 11003979

[23] Fulle S Protasi F Di Tano G Pietrangelo T Beltramin A Boncompagni S. The contribution of reactive oxygen species to sarcopenia and muscle ageing. Exp Gerontol. (2004) 39:17–24. 10.1016/j.exger.2003.09.012 14724060

[24] Kong SY Bostick RM Flanders WD McClellan WM Thyagarajan B Gross MD. Oxidative balance score, colorectal adenoma, and markers of oxidative stress and inflammation. Cancer Epidemiol Biomarkers Prev. (2014) 23:545–54. 10.1158/1055-9965.EPI-13-0619 24443405 PMC3959731

[25] Peterson MD Zhang P Choksi P Markides KS Al Snih S. Muscle weakness thresholds for prediction of diabetes in adults. Sports Med. (2016) 46:619–28. 10.1007/s40279-015-0463-z 26744337 PMC4863981

[26] Kang S Moon MK Kim W Koo BK. Association between muscle strength and advanced fibrosis in non-alcoholic fatty liver disease: a Korean nationwide survey. J Cachexia Sarcopenia Muscle. (2020) 11:1232–41. 10.1002/jcsm.12598 32638541 PMC7567158

[27] Parra-Soto S Pell JP Celis-Morales C Ho FK. Absolute and relative grip strength as predictors of cancer: prospective cohort study of 445,552 participants in UK Biobank. J Cachexia Sarcopenia Muscle. (2022) 13:325–32. 10.1002/jcsm.12863 34953058 PMC8818619

[28] Xu H Wang X Xiao W Xie Y Zhang X Xu S. Comparison between grip strength and relative grip strength in their relationship with allostatic load among adolescents. BMC Public Health. (2024) 24:2596. 10.1186/s12889-024-20129-0 39334007 PMC11430479

[29] Yang T Yi J He Y Zhang J Li X Ke S. Associations of dietary fats with all-cause mortality and cardiovascular disease mortality among patients with cardiometabolic disease. Nutrients. (2022) 14:3608. 10.3390/nu14173608 36079863 PMC9460477

[30] Kong SY Goodman M Judd S Bostick RM Flanders WD McClellan W. Oxidative balance score as predictor of all-cause, cancer, and noncancer mortality in a biracial US cohort. Ann Epidemiol. (2015) 25:256–62. 10.1016/j.annepidem.2015.01.004 25682727 PMC4369443

[31] Hernández-Ruiz Á García-Villanova B Guerra-Hernández E Amiano P Ruiz-Canela M Molina-Montes E. A review of a priori defined oxidative balance scores relative to their components and impact on health outcomes. Nutrients. (2019) 11:40774. 10.3390/nu11040774 30987200 PMC6520884

[32] Bull FC Al-Ansari SS Biddle S Borodulin K Buman MP Cardon G. World Health Organization 2020 guidelines on physical activity and sedentary behaviour. Br J Sports Med. (2020) 54:1451–62. 10.1136/bjsports-2020-102955 33239350 PMC7719906

[33] Tanabe Y Fujii N Suzuki K. Dietary supplementation for attenuating exercise-induced muscle damage and delayed-onset muscle soreness in humans. Nutrients. (2021) 14:10070. 10.3390/nu14010070 35010943 PMC8746365

[34] Besora-Moreno M Llauradó E Valls RM Tarro L Pedret A Solà R. Antioxidant-rich foods, antioxidant supplements, and sarcopenia in old-young adults ≥55 years old: a systematic review and meta-analysis of observational studies and randomized controlled trials. Clin Nutr. (2022) 41:2308–24. 10.1016/j.clnu.2022.07.035 36099667

[35] Aleksandrova K Koelman L Rodrigues CE. Dietary patterns and biomarkers of oxidative stress and inflammation: a systematic review of observational and intervention studies. Redox Biol. (2021) 42:101869. 10.1016/j.redox.2021.101869 33541846 PMC8113044

[36] Derbré F Gratas-Delamarche A Gómez-Cabrera MC Viña J. Inactivity-induced oxidative stress: a central role in age-related sarcopenia? Eur J Sport Sci. (2014) 14(Suppl. 1):S98–108. 10.1080/17461391.2011.654268 24444251

[37] Harman D. Aging: a theory based on free radical and radiation chemistry. J Gerontol. (1956) 11:298–300. 10.1093/geronj/11.3.298 13332224

[38] Lee HC Wei YH. Mitochondrial alterations, cellular response to oxidative stress and defective degradation of proteins in aging. Biogerontology. (2001) 2:231–44. 10.1023/a:1013270512172 11868898

[39] Jackson MJ McArdle A. Age-related changes in skeletal muscle reactive oxygen species generation and adaptive responses to reactive oxygen species. J Physiol. (2011) 589:2139–45. 10.1113/jphysiol.2011.206623 21320885 PMC3098693

[40] Zhang L Zalewski A Liu Y Mazurek T Cowan S Martin JL. Diabetes-induced oxidative stress and low-grade inflammation in porcine coronary arteries. Circulation. (2003) 108:472–8. 10.1161/01.CIR.0000080378.96063.23 12860917

[41] Schaap LA Pluijm SM Deeg DJ Harris TB Kritchevsky SB Newman AB. Higher inflammatory marker levels in older persons: associations with 5-year change in muscle mass and muscle strength. J Gerontol A Biol Sci Med Sci. (2009) 64:1183–9. 10.1093/gerona/glp097 19622801 PMC2759573

[42] Schaap LA Pluijm SM Deeg DJ Visser M. Inflammatory markers and loss of muscle mass (sarcopenia) and strength. Am J Med. (2006) 119:526. e9–17. 10.1016/j.amjmed.2005.10.049 16750969

[43] Li YP Schwartz RJ Waddell ID Holloway BR Reid MB. Skeletal muscle myocytes undergo protein loss and reactive oxygen-mediated NF-kappaB activation in response to tumor necrosis factor alpha. FASEB J. (1998) 12:871–80. 10.1096/fasebj.12.10.871 9657527

[44] Ramsey KA Rojer AGM D'Andrea L Otten RHJ Heymans MW Trappenburg MC. The association of objectively measured physical activity and sedentary behavior with skeletal muscle strength and muscle power in older adults: a systematic review and meta-analysis. Ageing Res Rev. (2021) 67:101266. 10.1016/j.arr.2021.101266 33607291

[45] Landi F Marzetti E Martone AM Bernabei R Onder G. Exercise as a remedy for sarcopenia. Curr Opin Clin Nutr Metab Care. (2014) 17:25–31. 10.1097/MCO.0000000000000018 24310054

[46] Evans WJ. Vitamin E, vitamin C, and exercise. Am J Clin Nutr. (2000) 72:647s−52s. 10.1093/ajcn/72.2.647S 10919971

[47] Schultchen D Reichenberger J Mittl T Weh TRM Smyth JM Blechert J. Bidirectional relationship of stress and affect with physical activity and healthy eating. Br J Health Psychol. (2019) 24:315–33. 10.1111/bjhp.12355 30672069 PMC6767465

[48] Parsons TJ Power C Manor O. Longitudinal physical activity and diet patterns in the 1958. Br Birth Cohort Med Sci Sports Exerc. (2006) 38:547–54. 10.1249/01.mss.0000188446.65651.67 16540844

[49] Villaverde P Lajous M MacDonald CJ Fagherazzi G Bonnet F Boutron-Ruault MC. High dietary total antioxidant capacity is associated with a reduced risk of hypertension in French women. Nutr J. (2019) 18:31. 10.1186/s12937-019-0456-0 31186024 PMC6560825

[50] Bai T Fang F Li F Ren Y Hu J Cao J. Sarcopenia is associated with hypertension in older adults: a systematic review and meta-analysis. BMC Geriatr. (2020) 20:279. 10.1186/s12877-020-01672-y 32762638 PMC7409686

