# Supplementary material for: The association between Dietary Oxidative Balance Score and muscle strength: NHANES 2011–2014
Source: Front Nutr. 2025 Jun 3;12:1563451. doi: 10.3389/fnut.2025.1563451 (PMC12172179; doi:10.3389/fnut.2025.1563451)
Supplement: Supplementary file 1 [file Table_1.docx]

Table S1 Dietary Oxidative balance score assignment scheme.

| **DOBS components** | Property | Scoring assignment | | |
| --- | --- | --- | --- | --- |
|  |  | 0 | 1 | 2 |
| Vitamin C (mg) | Anti-oxidant | Tertile 1 | Tertile 2 | Tertile 3 |
| Vitamin E (mg) | Anti-oxidant | Tertile 1 | Tertile 2 | Tertile 3 |
| Vitamin B6 (mg) | Anti-oxidant | Tertile 1 | Tertile 2 | Tertile 3 |
| Vitamin B12 (mg) | Anti-oxidant | Tertile 1 | Tertile 2 | Tertile 3 |
| Zinc (mg) | Anti-oxidant | Tertile 1 | Tertile 2 | Tertile 3 |
| Selenium (µg) | Anti-oxidant | Tertile 1 | Tertile 2 | Tertile 3 |
| Carotenoid (µg) | Anti-oxidant | Tertile 1 | Tertile 2 | Tertile 3 |
| Niacin (mg) | Anti-oxidant | Tertile 1 | Tertile 2 | Tertile 3 |
| Copper (mg) | Anti-oxidant | Tertile 1 | Tertile 2 | Tertile 3 |
| Dietary fiber (gm) | Anti-oxidant | Tertile 1 | Tertile 2 | Tertile 3 |
| Calcium (mg) | Anti-oxidant | Tertile 1 | Tertile 2 | Tertile 3 |
| Magnesium (mg) | Anti-oxidant | Tertile 1 | Tertile 2 | Tertile 3 |
| Total folate (mcg) | Anti-oxidant | Tertile 1 | Tertile 2 | Tertile 3 |
| Riboflavin (mg) | Anti-oxidant | Tertile 1 | Tertile 2 | Tertile 3 |
| Iron (mg) | Pro-oxidant | Tertile 3 | Tertile 2 | Tertile 1 |
| Total fat (gm) | Pro-oxidant | Tertile 3 | Tertile 2 | Tertile 1 |

Table S2 Linear regression of handgrip strength for dietary oxidative balance score and physical activity subgroups.

|  | Model 1 | |  | Model 2 | |  | Model 3 | |
| --- | --- | --- | --- | --- | --- | --- | --- | --- |
|  | β (95% CI) | *P* |  | β (95% CI) | *P* |  | β (95% CI) | *P* |
| Subgroup 1 (N=1,244) | 1.00(Ref) | - |  | 1.00(Ref) | - |  | 1.00(Ref) | - |
| Subgroup 2 (N=1,315) | -0.326(-0.434, -0.218) | **<0.001** |  | -0.106(-0.189, -0.024) | **0.013** |  | -0.083(-0.177, 0.012) | 0.086 |
| Subgroup 3 (N=1,222) | -0.572(-0.677, -0.467) | **<0.001** |  | -0.195(-0.273, -0.116) | **<0.001** |  | -0.161(-0.248, -0.075) | **0.001** |
| Subgroup 4 (N=1,094) | -0.783(-0.879, -0.687) | **<0.001** |  | -0.303(-0.370, -0.235) | **<0.001** |  | -0.245(-0.345, -0.145) | **<0.001** |
| Subgroup 5 (N=482) | -0.619(-0.771, -0.468) | **<0.001** |  | -0.341(-0.467, -0.215) | **<0.001** |  | -0.312(-0.424, -0.200) | **<0.001** |
| Subgroup 6 (N=766) | -0.925(-1.085, -0.766) | **<0.001** |  | -0.445(-0.565, -0.325) | **<0.001** |  | -0.363(-0.463, -0.263) | **<0.001** |
| Subgroup 7 (N=803) | -0.952(-1.087, -0.818) | **<0.001** |  | -0.370(-0.460, -0.280) | **<0.001** |  | -0.315(-0.414, -0.216) | **<0.001** |
| Subgroup 8 (N=957) | -1.130(-1.237, -1.022) | **<0.001** |  | -0.430(-0.517, -0.344) | **<0.001** |  | -0.318(-0.433, -0.202) | **<0.001** |

Subgroup 1 is the DOBS Q4 and the active group; Subgroup 2 is the DOBS Q3 and the active group; Subgroup 3 is the DOBS Q2 and the active group; Subgroup 4 is the DOBS Q1 and the active group; Subgroup 5 is the DOBS Q4 and the inactive group; Subgroup 6 is the DOBS Q3 and the inactive group; Subgroup 7 is the DOBS Q2 and the inactive group; Subgroup 8 is the DOBS Q1 and the inactive group; Model 1 without adjustments; Model 2 additionally adjusted for sex, age and race; Model 3 additionally adjusted for energy intake, cotinine level, alcohol consumption, education level, marital status, PIR, diabetes, hypertension and arthritis; *P*<0.05 was set as the threshold of statistical significance and marked in bold values.

Table S3 Linear regression of handgrip strength for dietary oxidative balance score and hypertension subgroups.

|  | Model 1 | |  | Model 2 | |  | Model 3 | |
| --- | --- | --- | --- | --- | --- | --- | --- | --- |
|  | β (95% CI) | *P* |  | β (95% CI) | *P* |  | β (95% CI) | *P* |
| Subgroup 1 (N=1,180) | 1.00(Ref) | - |  | 1.00(Ref) | - |  | 1.00(Ref) | - |
| Subgroup 2 (N=1,347) | -0.375(-0.488, -0.263) | **<0.001** |  | -0.141(-0.225, -0.058) | **0.002** |  | -0.099(-0.195, -0.003) | **0.043** |
| Subgroup 3 (N=1,313) | -0.579(-0.665, -0.492) | **<0.001** |  | -0.201(-0.260, -0.141) | **<0.001** |  | -0.160(-0.240, -0.080) | **<0.001** |
| Subgroup 4 (N=1,221) | -0.753(-0.852, -0.655) | **<0.001** |  | -0.287(-0.366, -0.208) | **<0.001** |  | -0.208(-0.319, -0.096) | **0.001** |
| Subgroup 5 (N=546) | -0.503(-0.621, -0.386) | **<0.001** |  | -0.333(-0.435, -0.231) | **<0.001** |  | -0.258(-0.348, -0.168) | **<0.001** |
| Subgroup 6 (N=734) | -0.830(-0.952, -0.707) | **<0.001** |  | -0.412(-0.500, -0.324) | **<0.001** |  | -0.297(-0.378, -0.215) | **<0.001** |
| Subgroup 7 (N=712) | -0.931(-1.069, -0.792) | **<0.001** |  | -0.375(-0.466, -0.285) | **<0.001** |  | -0.274(-0.368, -0.180) | **<0.001** |
| Subgroup 8 (N=830) | -1.246(-1.364, -1.129) | **<0.001** |  | -0.487(-0.573, -0.402) | **<0.001** |  | -0.315(-0.437, -0.193) | **<0.001** |

Subgroup 1 is the DOBS Q4 and the non-hypertension group; Subgroup 2 is the DOBS Q3 and the non-hypertension group; Subgroup 3 is the DOBS Q2 and the non-hypertension group; Subgroup 4 is the DOBS Q1 and the non-hypertension group; Subgroup 5 is the DOBS Q4 and the hypertension group; Subgroup 6 is the DOBS Q3 and the hypertension group; Subgroup 7 is the DOBS Q2 and the hypertension group; Subgroup 8 is the DOBS Q1 and the hypertension group; Model 1 without adjustments; Model 2 additionally adjusted for sex, age and race; Model 3 additionally adjusted for energy intake, cotinine level, alcohol consumption, education level, marital status, physical activity, PIR, diabetes and arthritis; *P*<0.05 was set as the threshold of statistical significance and marked in bold values.
